# Microwave Non-Destructive Testing for Delamination Detection in Layered Composite Pipelines

**DOI:** 10.3390/s21124168

**Published:** 2021-06-17

**Authors:** Przemysław Sobkiewicz, Paweł Bieńkowski, Wojciech Błażejewski

**Affiliations:** 1 Department of Telecommunications and Teleinformatics, Wroclaw University of Science and Technology, Janiszewskiego 9, 50–370 Wroclaw, Poland; Pawel.Bienkowski@pwr.edu.pl; 2 Department of Mechanics, Materials and Biomedical Engineering, Wroclaw University of Science and Technology, Smoluchowskiego 25, 50–370 Wroclaw, Poland; Wojciech.Blazejewski@pwr.edu.pl

**Keywords:** microwave imaging, non-destructive testing (NDT), delamination, microwave imaging probes, layered dielectric composites

## Abstract

Microwave imaging and defectoscopy are promising techniques for dielectric composite evaluation. Their most significant advantage is their relatively high penetration depth. Another feature worth noting is that traditional methods could not acquire an internal content with such a low impact on both the sample and surrounding environment, including the test operator, compared to other techniques. This paper presents microwave non-destructive and noninvasive methods for quality evaluation of layered composite materials using an open-ended waveguide probe. Pure |*S*_11_| parameters only exceptionally give a clear answer about the location of material cracks. Therefore, this makes it necessary to analyze these parameters simultaneously along with several other factors, such as stand-off distance, probe type or wave polarization. The purpose of the work was to find the dependency between the physical state of a layered composite powerplant pipeline and the S-matrix parameters response (reflection and transmission parameters) in a Ku frequency band that has not yet been extensively researched. Lower-frequency measurements broaden the application possibility for thicker composites, mainly because of a higher penetration depth and measurement setup availability. Different methods have been shown, including reflection and transmission/reflection methods, both in close proximity and in stand-off distance. The measurements are based on a low-complexity experimental setup.

## 1. Introduction

The number of composite materials surrounding us is growing at an above-average rate in many fields, e.g., automotive hydrogen tank and car chassis, avionics-fuselages, aircraft wings, or industrial applications—layered powerplant pipelines. Critical application areas make non-destructive testing of composite materials crucial for cost optimization, repeatability, and quality control throughout the whole product lifetime. Microwave imaging techniques are often applied in the noninvasive and non-destructive testing (NDT) of dielectric materials, due to their unique characteristics, such as high penetration depth, low measurement setup complexity, in-touch or stand-off operation zone, lack of requirement for a coupling agent, and environmental safety. Despite the mentioned advantages, microwave techniques also have limitations caused by the typical lack of a unique design of the waveguide probes, antennas, or even whole imaging setup for each specific application.

Propagated and radiated electromagnetic waves for NDT in microwave bands have been proposed for years. Due to the lossy nature of dielectric materials and the following internal dissipation, electromagnetic waves can heat, dry, or disinfect dielectric surfaces and structures. Many efforts have been made to use microwaves for retrieving information, such as material composition, and for imaging internal structures. Researches focus on imaging systems for biomedical applications, civil engineering, and industrial applications. It is worth noting that projects aimed at defining dielectric properties are conceptionally similar to microwave imaging methods [1].

However, many efforts have been made to detect composite cracks in the frequency bands adjacent to those used in this study [2,3]. Structural health monitoring of the wind turbine blades made of glass fiber-reinforced polymer (GFRP) composite, presented in [3], shows a possibility of crack detection with a near-field waveguide probe, in the frequency range 18–19.5 GHz. A similar inspection method is microwave holography [4]. Samples were examined for hidden flaws presence in composite polyurethane-based thermal insulation coatings obtaining promising results for frequencies between 20 and 24 GHz.

Despite these and other preliminary developments, the current state-of-the-art measurement techniques still suffer from a lack of delamination detection measurements in the K band, particularly in the Ku band [2,5]. To the author’s best knowledge, little work has been conducted on the near-field microwave delamination detection in the composite powerplant pipeline constructions. Accurate results for hidden flaws inspection will be achieved when the wavelength is comparable to the sample size [3]. Other factors, such as measurement apparatus availability, costs, and a relatively simple probe design process, make Ku and K bands promising for this specific application of composite pipelines, and should be investigated further. Nowadays, despite many advantages, commercially proposed methods apply to a limited extent as the number of layers, inclusions, and the thickness of composite structures increase.

For the sake of safety and non-ionizing nature, ultrasounds are widely used not only in medical imaging, but also in non-destructive testing. Several authors have proposed defect localization methods, based on acoustic and ultrasonic sensing, by installing transducers on structures (in-touch form) [6,7,8]. Proper imaging modalities allow flaws detection of composite materials—however, strong signal attenuation and scattering effects limit its applicability. It is caused by high density, high fiber content, and often, in-touch measurement requirements [1].

Satisfactory results are achieved with tomography, but many application and safety aspects are concerned. Using a scanning electron microscope with a high resolution gave good results, but it is limited to scanning only the outer surface. It is also still considered a relatively expensive solution [9].

A comprehensive comparison of the above methods was shown in [9]. The authors demonstrate the sensitivity of a coupled spiral inductor sensor for impact detection in carbon fiber composites, for radio frequencies 10–1000 MHz.

In this context, the usage of broadband microwave testing in Ku (12–18 GHz) and K (18–26.5 GHz) bands is considered a promising way to bypass several other methods limitations [10]. Electromagnetic waves are susceptible to changes associated with boundary interfaces. The depth of wave penetration can reach a few centimeters. The main values are low energy level of non-ionizing radiation, the nonsignificant impact on the electromagnetic environment, material under test (*MUT*) and operator, the potentiality to focus electromagnetic energy and the low-complexity of the measurement setup. Microwaves, due to having indisputably low invasiveness, are significant interests for the purposes of composite inspection for the whole lifetime cycle [5,11,12]. It has also been suggested [5,10,13,14] to merge different NDT methods if it is only possible to conduct them simultaneously.

### 1.1. Near-Field Imaging Technique

Microwave imaging techniques are based on short-range measurement systems that are able to retrieve material properties because of the interaction between the investigated waves (incident and received) and the target material under test. Information is often contained in the scattering parameters, that is, the reflection coefficients *S*_11_, *S*_22_, and the transmission coefficients *S*_12_ and *S*_21_. Depending on the retrieved parameters, the non-resonant measurements are divided into reflection and transmission concepts [15]. Open-ended rectangular waveguides are quite often used as scanning probes in near-field imaging systems, due to their possibility to work in a broad frequency range, their high resolution, and their relatively inexpensive cost [16].

**Figure 1:**
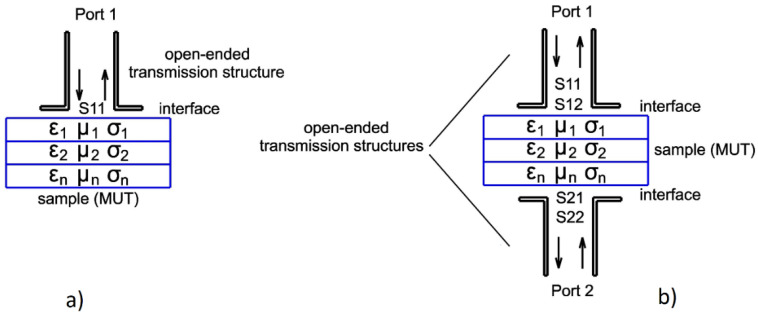
(**a**) Open-ended waveguide reflection setup for reflection measurement; (**b**) open-ended waveguide setup for transmission/reflection measurement.

In a reflection method, the *MUT* is inserted into a particular position—mainly at the open-ended transmitting/receiving probe interface. Due to material absorption, the *MUT* causes a change in the impedance load, and, in turn, the incident wave reflects and dissipates. The internal properties of the sample are derived because of the wave reflection and impedance discontinuity caused by, e.g., the presence of inclusion, delamination, cracks, or the connection of different types of layers—presented in Figure 1a.

The transmission method obtains information from a sample inserted between the transmitting and receiving probes. The mentioned setup can be made of two transmission lines, such as rectangular waveguides, with the *MUT* between their apertures—presented in Figure 1b. All four scattering parameters can be measured. Other factors, such as the *MUT* length and depth or the relevant position of the two reference planes, should be considered [5].

### 1.2. Delamination Crack Characteristics

Delamination is crucial damage for composite with layers adhesively bonded together. Damage occurs after impact or high-pressure exposure affecting the structural integrity between adjacent layers. Therefore, it is a standard failure mode in stratified composites. Significant damage can occur on both the outer/inner surfaces, or inside the material [17,18].

Problem complexity arises when invisible delamination occurs under the outer pipeline shell and no part of the failure can be found by the surface’s non-destructive testing methods (e.g., visual inspection, liquid penetrate inspection, eddy currents). The case mentioned above can be handled only using limited non-destructive testing methods [1].

### 1.3. Goals

This work aims to validate the possibility of crack detection in an examined frequency range beyond the state-of-the-art, in terms of the current microwave measurement capabilities. Further research will be focused on searching for the dependency between the physical state (and, as a result, mechanical strength) of dielectric layered composite powerplant pipelines, with S-matrix parameters obtained by microwave inspection methods. The measurements are based on reflection and transmission/reflection concepts. An accurate material evaluation based on the method’s comparison and proposition of particular applications, including in-touch and stand-off variants, has been presented. A low-complexity experimental setup was built. The presented paper is arranged as follows: paragraph two includes the experimental setup presentation, the third paragraph deals with the results, and the last paragraph contains the conclusions on the effectiveness, the applicability of the method, and the significance of the measurements.

## 2. Materials and Methods

### 2.1. Open-Ended Waveguide Defectoscopy Measurement System

**Figure 2:**
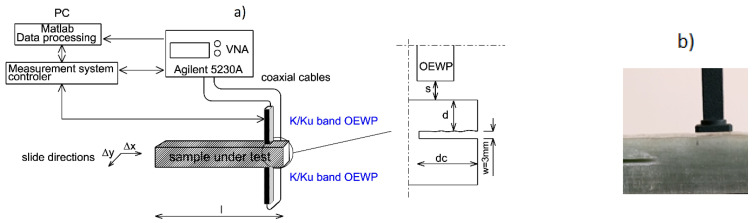
(**a**) Transmission measurement setup scheme with open-ended waveguide probes. (**b**) Open-ended waveguide probe located over the sample. Where s—stand-off distance, d—delamination depth, dc—delamination crack length, w—delamination width.

The measurement setup is schematically shown in Figure 2, and it consists of a 4-port vector network analyzer (Agilent N5230A) connected via Sucoflex microwave cables to rectangular waveguide probes. VNA works in a frequency-sweeping mode to reveal the defects at specific frequencies by getting multiple frequency resonances. Probes scan in stand-off distance(s) over the top face of the sample storing the |*S*_11_|and |*S*_22_| reflection coefficient, and |*S*_12_|and |*S*_21_| transmission coefficient. The probes were assembled at a fixed position to avoid phase shifts because of cable movements. Subsequently, *MUT* was placed on a plate slider mounted on scan guides. Slider moves in x–y directions with step size Δx = Δy = 2 mm, while probes inspect the surface. Decreasing the step size does not affect the defectoscopy quality much; however, it will significantly increase the image data processing and measurement time.

Referring to transmission line theory, the reflection coefficient can be described as follows:

(1) $$\Gamma =\Gamma e^{j\varphi }=\frac{Z_{MUT}-Z_{OEWP}}{Z_{MUT}+Z_{OEWP}}\;$$

where *Z_MUT_* is the intrinsic impedance of the material under test and *Z_OEWP_* is the open-ended rectangular waveguide characteristic impedance for the dominant mode. In this study, an *OEWP* with TE_10_ dominant mode is used.

(2) $$Z_{MUT}=\sqrt{\frac{{µ}_{0}{µ}_{r}}{\varepsilon_{0}\varepsilon_{r}}}$$

(3) $$Z_{OEWP}=\frac{\omega \mu }{\beta }=K\frac{\lambda_{g}}{\lambda }=\frac{K}{\sqrt{1-\eta^{2}}}$$

where $K=\sqrt{µ/\varepsilon }$ and *η* = *λ*/2a, a is the transverse width, *β* is the phase constant, and *λ_g_* is the guide wavelength [19,20].

According to the theory of multiple reflections, the S-matrix parameters of the finite length sample can be written as a function of the reflection coefficient at the interface of two infinite media *Γ*, and as a function of the phase factor *T*.

(4) $$S_{11}=R_{1}^{2}\;\frac{\Gamma \left(1-T^{2}\right)}{1-\Gamma^{2}T^{2}}$$

(5) $$S_{21}=\frac{\left(1-\Gamma^{2}\right)T}{1-\Gamma^{2}T^{2}}$$

where *T* is a phase factor, *R*_1_ is the reference plane transformations. Nicolson, Ross and Weir combined Equations (4) and (5) (NRW equation) for *S*_11_ and *S*_12_, deriving formulas for permittivity and permeability calculations [21].

(6) $$\Gamma =K\mp \sqrt{K^{2}-1}$$

(7) $$K=\frac{\left(S_{11}^{2}-S_{21}^{2}\right)+1}{2S_{11}}$$

(8) $$T=\frac{\left(S_{11}+S_{21}\right)-\Gamma }{1-\left(S_{11}+S_{21}\right)\Gamma }$$

The correct sign in Equation (6) should be chosen according to the requirement that $\left|\Gamma \right|\leq 1$ [12].

(9) $${µ}_{r}=\frac{1+\Gamma }{\left(1-\Gamma \right)\Lambda \sqrt{\left(1\;/\;\lambda_{0}^{2}\right)-\left(1\;/\;\lambda_{c}^{2}\right)}}$$

(10) $$\varepsilon_{R}=\frac{\lambda_{0}^{2}}{{µ}_{r}\left[\left(1\;/\;\lambda_{c}^{2}\right)-\left(1\;/\;\Lambda^{2}\right)\right]}$$

where *λ*_0_ is the free space wavelength, *λ_c_*—waveguide cut-off frequency. The angle between the electrical field vector E of the incident electromagnetic wave and the *MUT* fiber arrangement also affects the reflection coefficient. Delamination cracks cause an essential change in *MUT* impedance *Z_IN_*. The idea of a qualitative measurement setup is that there is no need to know the proper dielectric parameters of the material. This is especially important for composite materials where a lack of material notes is common. Therefore, non-resonant dielectric parameters estimation methods have been adopted to estimate dielectric parameters and ensure that the material is lossy enough. The presented qualitative method works on dielectric parameter differences rather than exact values. Transparent and low-loss material causes undetectable reflections between composite and crack layers. On the other hand, conductive materials limit penetration depth to few micrometers. According to NRW algorithm, relative permittivity of a sample is equal to $\varepsilon_{R}=7-9$ in the 15–20 GHz range. In all calculations, the conductivity is taken to be σ=1.5 mS/m, which corresponds to the dielectric properties of GFRP. The assumption that material is nonmagnetic was made by $\mu =\mu_{0}$.

Depth of defect detection based on electromagnetic waves is limited by skin effect and corresponding penetration depth δ, which is given by the following:

(11) $$\delta =\frac{1}{\sqrt{\pi f\mu \sigma }}$$

where *f*—frequency (Hz), μ—permeability (H/m), σ—susceptibility (S/m).

For measurement, frequency band, penetration depth reaches 12.0 cm inside *MUT*, and the real penetration depth can vary because of receiver sensitivity. The primary way to detect crack or delamination in composite material is S-matrix parameter measurements—searching for reflection and transmission parameters at a given reference plane. Material parameters are obtained because of impedance changes in different layers interfaces and propagating speed change of EM wave in the dielectric medium [22]. The proposed method gives good results for material that absorbs and reflects EM waves.

Specific frequencies are more sensitive to material flaws and can reveal the cracks more finely. These frequencies differ depending on *MUT* material, delamination shape, depth (d), and width (w). VNA works in a broad spectrum (frequency-sweeping mode) to get frequency resonances, and thus revealing the defects at particular frequencies. These frequencies are selected by calculating reflection coefficient variations in all sensing positions on scanned surfaces. Certain frequencies with the highest variance should be investigated as candidates. It should be noted that correct inspection frequency candidates choices can only be made case by case [3,23,24,25].

### 2.2. Pipeline Composite Samples

Tested samples are dismantled parts from power plant “Elektrociepłownia Opole”. They are sections of the composite pipeline intended for cooling water systems. The material was made by winding glass filaments soaked in polyester resin under tension over a rotating mandrel. Fiber layers are arranged together alternately at a 45-degree angle, assuming the same dielectric parameters except for the delamination layer. There is no other boundary phase between the layers as the material is homogeneous with the same proportions of glass fibers and resin (50% volume of each phase) in the thickness of the tested material.

A set of samples purposely incised delamination defects of different depths have been measured—collectively shown in Table 1. All pieces have been chosen accordingly, assuming that the sample thickness should be much larger than the spin of the rectangular waveguide probe aperture.

**Figure 3:**
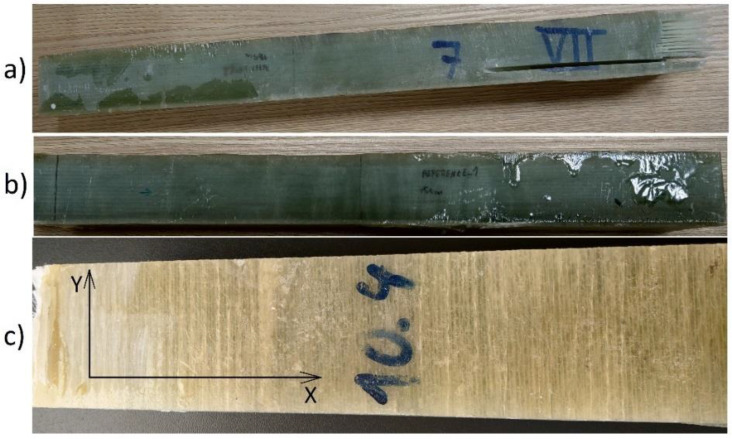
Exemplary pipeline composite bars (**a**) delamination d = 1.0 cm; (**b**) reference sample; (**c**) sample surface.

Each sample was incised at a different delamination depth. The size of the crack is the same for all samples and ends at 6.0 cm from the right face—shown in Figure 2. Constant 3.0 mm crack width was chosen because it is an average delamination width of a dismantled pipeline. Figure 3 presents a few exemplary samples.

Sample numbers 1–25 were incised to imitate delamination cracks. Incisions were made parallelly to the scanning surface, and they start at one end; incision lengths for all bars are the same. The width (w) of the defect is also the same in all cases and is equal to 0.3 cm. Sample numbers 26–30 were used as a reference and had no incision at all.

## 3. Results and Discussion

These measurements aimed to investigate the frequency candidates that are sensitive to delamination cracks. It was found that for different measurement types, the most sensitive frequencies belong to two ranges—the first starts at 15.4 and ends at 16.9 GHz, and the second one from 18.5 to 19.0 GHz. As one-port and two-port measurements, reflection coefficients |*S*_11_| and |*S*_22_|, or transmission coefficients |*S*_12_| and |*S*_21_| are acquired from the vector network analyzer. The measured S-parameters data are shown in subsection figures below. Table 2 presents a short review of the measurement setup parameters. To evaluate the sensitivity for both of the measurement types, line scans were performed outside the samples to detect delamination, as shown in Figure 3. The asymmetrical footprint in the x-y plane of the rectangular probe results in the sensitivity and imaging resolution becoming a function of E-field orientation. The probe aperture was oriented such that the electric field vector was orthogonal to the delamination edge to provide a higher resolution. It can be observed that the images produced by the probe indicate S11 strength corresponding to delamination depth.

### 3.1. Reflection Measurements

**Figure 4:**
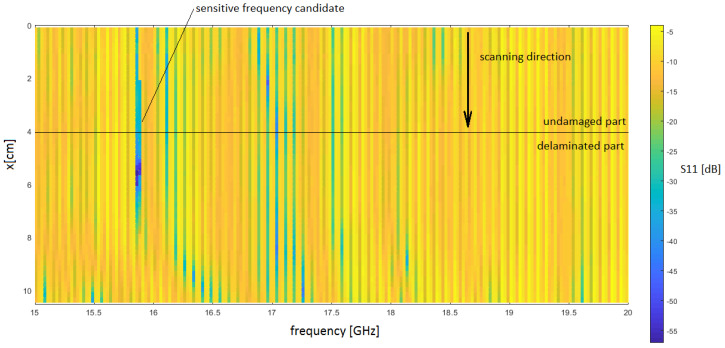
Exemplary of measured reflection (|*S*_11_|) parameter over the lengthwise OX cross-section of sample number 17 for the frequency range from 15 to 20 GHz. Incision depth, d—1.0 cm; stand-off distance, s—0.1 cm.

The reflection measurement was carried in two cases. At the initial stage, the sample was placed on a conducting plate holder and moved over the scan region under the OEWP. Subsequently, S-matrix parameters were monitored for every single step in the x and y direction. After that, the exact broadband measurements were repeated without the plate—the sample was placed directly on a plastic holder—a 3D example of the reflection *|S*_11_| parameters for the OX cross-section is shown in Figure 4 below. The delamination edge starts at 4.0 cm. It was observed that the peak value occurs when the OEWP remains in position above the delamination edge.

**Figure 5:**
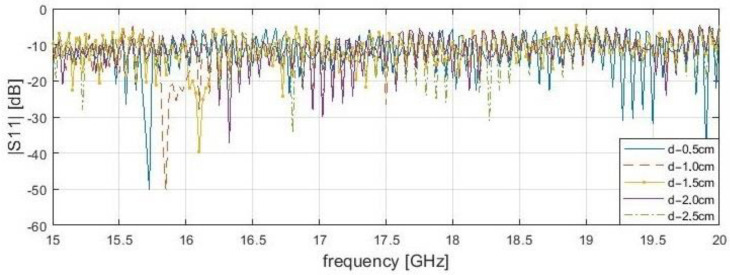
Measured reflection (|*S*_11_|) of bar samples without conductive plate behind at a position of delamination start (x—4.0 cm) for the frequency range from 15 to 20 GHz with stand-off distance, s—0.1 cm.

**Figure 6:**
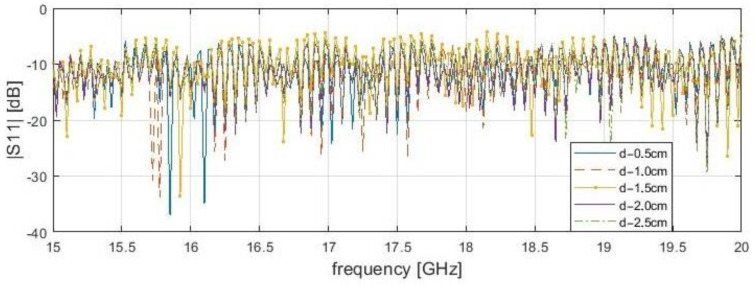
Measured reflection (|*S*_11_|) of bar samples with a conductive plate behind at a position of delamination start (x—4.0 cm) for the frequency range from 15 to 20 GHz with stand-off distance, s—0.1 cm.

**Figure 7:**
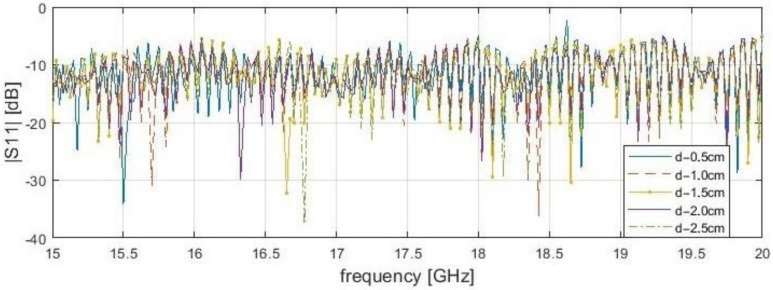
Measured reflection (|*S*_11_|) of bar samples without conductive plate behind at a position of delamination start (x—4.0 cm) for the frequency range from 15 to 20 GHz with stand-off distance, s—2.0 cm.

**Figure 8:**
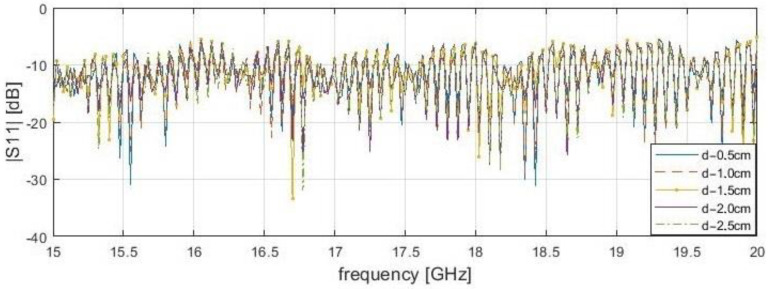
Measured reflection (|*S*_11_|) of bar samples with a conductive plate behind at a position of delamination start (x—4.0 cm) for the frequency range from 15 to 20 GHz with stand-off distance, s—2.0 cm.

It is worth noting that dependence of resonance frequency and the delamination depth has been noticed (Figure 5, Figure 6, Figure 7 and Figure 8). It can be observed that for s = 0.1 cm, the |*S*_11_| resonance curve tends to become flattered as the delamination depth and frequency increase. For this reason, detection becomes more difficult for delamination under 2.5 cm beneath the scanning surface. It was found that the optimal measurement frequency for the reflection method in close proximity could be determined in a range of 15.4 to 16.4 GHz (for a delamination depth limit up to 2.5 cm). A summary of the presented reflection method results is shown in Table 3 below. The reference level is established as the average |*S*_11_| value over the entire frequency range for the delamination start point. The threshold level is defined as the μ−3σ, where μ—average magnitude for samples before the incision, and σ—standard deviation. The conducting plate does not change the resonance frequency candidates, but strongly affects the reflection S-parameters magnitudes. In both cases, for 2.0 cm, the stand-off distance results do not give a clear answer about the frequencies for investigation, because many results are near the threshold, unlike for s = 0.1 mm.

|*S*_11_| dip frequencies are changing due to the variance of relative dielectric constant for samples with different delamination depths. Further, |*S*_11_| tends to diminish with increasing incision depth, and an example for this is as follows: stand-off, 0.1 cm—about 7 dB/1 cm without CP, about 3 dB/0.1 cm with CP. According to [1], PEC can provide better sensitivity, but in this case, the samples are too thin, producing, in turn, significant background noise. A direct connection between the S-matrix parameters, crack parameters, and peak frequencies will be shown in further works.

### 3.2. Transmission Measurements

**Figure 9:**
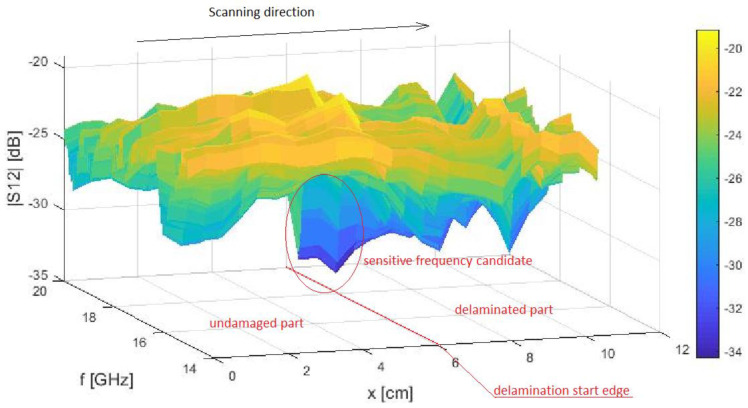
Exemplary measured transmittance (|*S*_12_|) over the lengthwise OX cross-section for the frequency range from 15 to 20 GHz. Standoff distance, s—0.1 cm. Sample number 23 with incision depth, d—1.0 cm.

The measurements were prepared with the use of two identical open-ended waveguide probes, mounted symmetrically on both sides of the *MUT* (schematically presented in Figure 1b). The stand-off distances were the same for both of the probes. Afterward, a sample was placed directly on a plastic holder, and the S-matrix parameters were monitored for every single step. An example of a 3D result of |*S*_21_| parameters for two samples, with d = 1.0 cm and d = 0.5 cm, is shown in Figure 9. A summary of the presented transmission method results is shown in Table 4.

**Figure 10:**
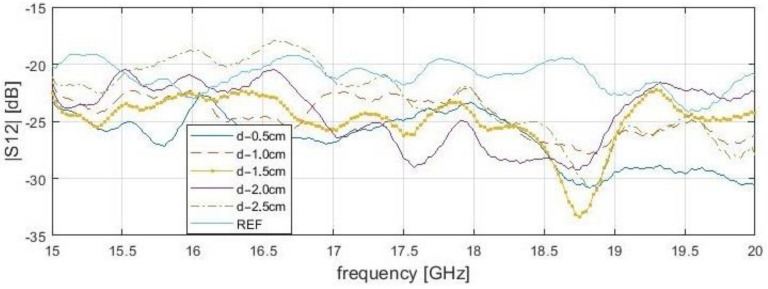
The transmittance (|*S*_12_|) of bar samples at a position of delamination start (x—4.0 cm, y—1.0 cm) for the frequency range from 15 to 20 GHz. Stand-off distance, s—0.1 cm.

**Figure 11:**
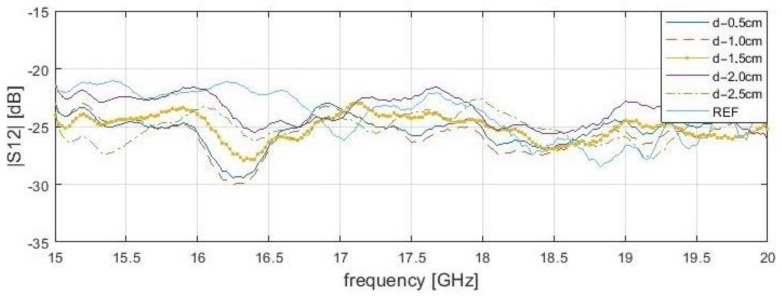
The transmittance (|*S*_12_|) of bar samples at a position of delamination start (x—4.0 cm, y—1.0 cm) for the frequency range from 15 to 20 GHz. Stand-off distance, s—2.0 cm.

Only one lengthwise cross-section result was presented in both of the analyzed situations—reflection and transmission measurements. The examined S-matrix parameters changes are also valid for any other analyzed cross-section of a sample. Unlike the reflection results, the transmittance parameters in close proximity are shifted to a higher frequency range of 18.6 to 19 GHz (Figure 10 and Figure 11). Setting the probe above the delamination edge causes |*S*_12_| minimum peaks for a specific frequency. Therefore, distinguishing delamination at different depths should be based on the frequency peak rather than on the S-parameters magnitudes, because of high magnitude variance. Surface roughness causes wave dispersion, and this impact depends on the surface texture angle of the wave incident and frequency. According to the Rayleigh criterion, it is assumed that the surface is flat for the measurement range. Transmission methods are more sensitive for deep delamination compared with reflection methods. Additionally, transmission methods are susceptible to even slight probe displacement. On the other hand, there is a relatively wide |*S*_11_| peak area caused by the large rectangular probe sidelobe levels compared to other probe types, such as the coaxial line probe. This explains the image deterioration and edge shift for reflection and transmission cases (|*S*_11_| peak begins before probe aperture is set directly above delamination edge). However, “in vivo” transmission measurement applications for powerplant pipelines are limited.

## 4. Conclusions

Despite plenty of conducted researches regarding non-destructive testing methods used in material engineering in general, there is still relatively little work done on composite materials. The presented paper shows two microwave defectoscopy methods successfully employed to detect delamination in the specific type and family of fiberglass composite with the resin binder used for powerplant pipeline constructions. According to the authors’ best knowledge, such research has not yet been conducted on this specific type of specimen.

Depending on the technique used and the delamination depth, the most sensitive S-parameter changes were observed in the inspection frequencies from 18.6 to 19.0 GHz, which narrows down the frequency spectrum found by other researchers.

Except that, a new sensitive frequency range for crack detection has been found, which is 15.4–16.9 GHz. It has a better penetration depth and measurement apparatus availability.

A conducting plate (CP) does not change the resonant frequency; also, a higher SNR can be achieved without a CP. For this sample type, the additional CP placed behind the model does not emphasize frequency candidates other than those revealed without it.

For the reflection method (without CP, stand-off = 0.1 mm), promising results were obtained; the |*S*_11_| magnitudes were significantly lower than the established threshold, opening the possibility of deeper detection in future works. For the transmission methods, increasing the stand-off distance to 2.0 cm reduces sensitivity substantially, but it can be seen that absorption in 15.4–16.9 GHz occurs.

Inconsiderable inaccuracy of frequency determination can be caused by unideal surface smoothness, which influences the probes stand-off distance. Considering the resonant frequency, and |*S*_11_|or |*S*_12_|, the magnitude gives comprehensive information about the target defect. More extensive frequency changes were observed when the stand-off distance increased; then revealing the weakness became more complex, and this creates a need for further researches. It has been shown that high magnitude variance makes using only magnitude parameters insufficient for delamination depth estimation.

The proposed method allows quick crack detection of relatively small samples, with a satisfying sensitivity. Due to the vastness of composite material types, shapes, and corresponding defects, the presented work can be retargeted and adapted for other specific environments. The proposed method may prove to be a solution to the need of the industry to apply a product quality control measure at the output of the production process (pultrusion or winding). Until now, the quality control consisted of the detection of mainly surfaces defects and destructive tests carried out on representative samples. The use of the method described in the work, after its prior adaptation to industrial conditions, would allow avoiding waste from destructive tests and provide total quality control of all products. This would be justified, especially in the case of products that are critical system parts (such as the tested composite pressure pipeline or hydrogen tanks).

Despite the potential of the presented methods, they also have some limitations. In the course of the research, it was found that it is possible to detect cracks up to 3.0 mm in width. Further works will be focused on the sensitivity of detection at different widths of incisions and their possible fluid fillings.

The presented work should be considered as preliminary communication, intended to be a qualitative indication of the delamination boundary.

## Figures and Tables

**Table 1:** List of composite samples for reflection and reflection/transmission measurements.

Sample Number	Incision Depth (d) [cm]	Sample Number	Incision Depth (d) [cm]
1–5	2.5	16–20	1.0
6–10	2.0	21–25	0.5
11–15	1.5	26–30	Reference-no incision

**Table 2:** Reflection and transmission measurement parameters.

Parameter	Value
Sample size	width: 60 mm, height: 40–45 mm, length: 350–500 mm
Effective scan area	20 mm × 120 mm on every sample surface
Probe dimensions	aperture: width: 10.7 mm, height: 4.3 mm with a flange 23 × 23 mm (show in Figure 2b)
Δx, Δy	2 mm, 2 mm
Frequency band	15 GHz–20 GHz
Number of linear frequency points	401
Stand-off distance	0.1 cm and 2.0 cm

**Table 3:** Average reflection |*S*_11_| peak results for reflection method on frequency range 15–20 GHz for both of the following cases: without and with conducting plate.

Incision Depth	Without Conducting Plate					
	Reflection Method Stand-Off = 0.1 cm			Reflection Method Stand-Off = 2.0 cm		
	*f*_1_ [GHz]	|*S*_11_|_min_ [dB]	Threshold 3σ [dB]	*f*_2_ [GHz]	|*S*_11_|_min_ [dB]	Threshold 3σ [dB]
Average reference level (μ)	-	−17.0	−30.2	-	−13.7	−21.7
d = 0.5 cm	15.65	−48.2		15.50	−32.2	
d = 1.0 cm	15.85	−46.0		15.70	−29.2	
d = 1.5 cm	16.10	−38.7		15.40	−25.2	
d = 2.0 cm	16.22	−36.2		16.30	−27.9	
d = 2.5 cm	16.80	−34.5		16.60	−30.1	
Incision depth	With conducting plate					
	Reflection method standoff = 0.1 cm			Reflection method standoff 2.0 cm		
Average reference level (μ)	-	−14.1	−20.3	-	−12.9	−20.7
d = 0.5 cm	15.78	−34.2		15.55	−31.9	
d = 1.0 cm	15.90	−34.1		15.70	−29.2	
d = 1.5 cm	16.00	−31.7		16.70	−32.3	
d = 2.0 cm	16.10	−31.0		16.00	−29.8	
d = 2.5 cm	16.40	−28.4		16.77	−30.4	

**Table 4:** Average transmittance |*S*_12_|peak results for transmission method in the frequency range 15–20 GHz. σ—standard deviation of the average |*S*_12_| of healthy samples.

Incision Depth	Transmission Method Stand-Off = 0.1 cm			Transmission Method Stand-Off = 2.0 cm		
	*f*_1_ [GHz]	|*S*_12_|_min_ [dB]	Threshold 3σ [dB]	*f*_2_ [GHz]	|*S*_12_| _min_ [dB]	Threshold 3σ [dB]
Average reference Level (μ)	-	−20.7	−25.5	-	−24.2	−30.1
d = 0.5 cm	18.98	−31.8		16.35	−33.0	
d = 1.0 cm	18.79	−28.9		16.25	−32.9	
d = 1.5 cm	18.70	−31.4		16.31	−31.0	
d = 2.0 cm	18.68	−27.5		16.45	−30.7	
d = 2.5 cm	18.78	−29.2		16.40	−32.0	

## Data Availability

Not applicable.

## Abbreviations

Following abbreviations are used in this article:

CP	Conducting plate
EM	Electromagnetic
EME	Electromagnetic environment
*MUT*	Medium under test
NDT	Non-destructive testing
OEWP	Open-ended waveguide probe
VNA	Vector network analyzer
